# Expression of FAM83H and ZNF16 are associated with shorter survival of patients with gallbladder carcinoma

**DOI:** 10.1186/s13000-020-00985-1

**Published:** 2020-05-27

**Authors:** Sung Woo Ahn, Ae-Ri Ahn, Sang Hoon Ha, Usama Khamis Hussein, Jae Do Yang, Kyoung Min Kim, Ho Sung Park, See-Hyoung Park, Hee Chul Yu, Kyu Yun Jang

**Affiliations:** 1 Department of Surgery, Jeonbuk National University Medical School, Jeonju, Republic of Korea; 2 Department of Pathology, Jeonbuk National University Medical School, 567 Baekje-daero, Dukjin-gu, Jeonju, 54896 Republic of Korea; 3 Division of Biotechnology, Jeonbuk National University, Iksan, Republic of Korea; 4 Faculty of Science, Beni-Suef University, Beni-Suef, Egypt; 5 Department of Bio and Chemical Engineering, Hongik University, Sejong, Republic of Korea; 6 Research Institute of Clinical Medicine of Jeonbuk National University-Biomedical Research Institute of Jeonbuk National University Hospital, Jeonju, Republic of Korea

**Keywords:** Gallbladder, Carcinoma, FAM83H, ZNF16, Prognosis

## Abstract

**Background:**

Recently, FAM83H was reported to have roles in cancer progression in conjunction with oncogenic molecules such as MYC and b-catenin. Moreover, the data from the public database indicates a molecular relationship between FAM83H and zinc finger proteins, especially between FAM83H and ZNF16. However, studies on FAM83H and ZNF16 in gallbladder cancer have been limited.

**Methods:**

This study investigated the expression of FAM83H and ZNF16 in 105 gallbladder carcinomas.

**Results:**

In human gallbladder carcinomas, immunohistochemical expression of FAM83H was significantly associated with ZNF16 expression. In univariate analysis, nuclear and cytoplasmic expression of FAM83H or ZNF16 were significantly associated with shorter survival of gallbladder carcinoma patients. Multivariate analysis revealed the nuclear expression of FAM83H as an independent indicator of poor prognosis of overall survival (*p* = 0.005) and relapse-free survival (*p* = 0.005) of gallbladder carcinoma patients. Moreover, co-expression patterns of nuclear FAM83H and ZNF16 were also independent indicators of shorter survival of gallbladder carcinoma patients (overall survival; *p* <  0.001, relapse-free survival; *p* <  0.001).

**Conclusions:**

This study suggests FAM83H and ZNF16 are associated with the progression of gallbladder carcinoma, and the expressions of FAM83H and ZNF16 might be novel prognostic indicators of gallbladder carcinoma patients.

## Background

FAM83H is primarily known for its importance in tooth development because mutation in FAM83H causes amelogenesis imperfecta [1, 2]. However, recent reports have shown various roles of FAM83H in both normal cells and cancer cells. In addition to the role of FAM83H in enamel formation in teeth, it is important in maintaining the intracellular actin filament framework and is involved in cancer progression [3–6]. Expression of FAM83H in cancer cells is elevated compared with normal cells, which suggests FAM83H plays a role in tumorigenesis [7]. Disruption of the actin filament network by deregulated FAM83H expression is thought to induce epithelial-to-mesenchymal transition (EMT) [3]. FAM83H-mediated stimulation of EMT accelerates cancer progression [5, 6]. Moreover, FAM83H stimulates the proliferation of cancer cells by inducing cell cycle progression in conjunction with MYC and canonical Wnt pathways [5, 6]. The oncogene *MYC* transcriptionally stimulates FAM83H expression, and consequently, FAM83H stabilizes β-catenin to activate the canonical Wnt pathway [5]. In kidney cancer, FAM83H regulates the expression of PANX2 [8]. Therefore, it is likely that there is a more complex molecular network involved in FAM83H-associated tumorigenesis.

Zinc finger proteins have pleiotropic roles as transcription factors in cellular processes [9, 10]. There are several types of zinc finger proteins classified according to their molecular structure: C2H2-, ring-, PHD-, and LIM-type [10]. Among them, the C2H2-type is the largest group of zinc finger proteins, of which ZNF16 (HZF1) is a member [10, 11]. Zinc finger proteins have diverse roles in normal physiology and tumorigenesis [9, 10]. Some zinc finger proteins are tumorigenic, and others are tumor-suppressive [9]. It has been reported that ZNF16 has a function in the differentiation of erythroid cells and megakaryocytes [11]. However, the role of ZNF16 in human cancer is not clear. Despite limited reports on the role of ZNF16 in tumorigenesis, data in the public database indicates that ZNF16 might have a role in tumorigenesis. ZNF16 expression is higher in cancers compared with normal cells in the breast, gastrointestinal tract, lung, ovary, and hepatobiliary tract (cBioPortal database; http://www.cbioportal.org. Accessed 2 March 2020) [12, 13]. In addition, ZNF16 is the molecule that has the most significant correlation with FAM83H in the cholangiocarcinoma (cBioPortal and GEPIA database; http://gepia.cancer-pku.cn. Accessed 2 March 2020) [12–14]. Therefore, it has been suggested that FAM83H and ZNF16 might be involved co-operatively in tumorigenesis.

Gallbladder cancer comprises 1.2% of new cancer development and 1.7% of cancer death, worldwide [15]. The high incidence of gallbladder cancer has been reported in eastern Asia and southern America [16]. Gallbladder cancer is commonly correlated to inflammation, and inflammation-associated accumulation of genetic alteration is one of the main causes of gallbladder cancer development [17]. In addition, the inflammation-associated C2H2 zinc finger protein MAZ (Myc-associated zinc finger) stimulates cancer development [18]. Therefore, based on the possible relationship between FAM83H and ZNF6 in cancers of hepatobiliary sites, we investigated the expressions and prognostic significance of FAM83H and ZNF16 in human gallbladder cancers.

## Methods

### Human gallbladder carcinoma patients

This study included gallbladder carcinoma patients who operated on between January 2000 and December 2008. In total, 105 cases of gallbladder carcinoma for which histologic slides and paraffin-embedded tissue blocks were available were included in this study. The medical records and histologic slides were reviewed to obtain clinicopathological information. There were no patients who received neoadjuvant chemotherapy. Twenty-three patients received postoperative chemotherapy, and six patients received postoperative radiotherapy. Five patients received both adjuvant chemotherapy and radiotherapy. The clinicopathological factors evaluated in this study were the age of the patients, sex, preoperative levels of CEA and CA19–9 on serum, TNM tumor stage, T category of the tumor stage, lymph node metastasis, distant metastasis, lymphovascular invasion, histologic type, and histologic grade of cancer. Histologic factors and TNM stage of all cases were reviewed according to the WHO classification [17] and the 8th edition of the American Joint Committee Cancer Staging System [19]. This study was approved by the institutional review board of Jeonbuk National University Hospital (IRB number, CUH 2019–11-041) and was performed in compliance with the Declaration of Helsinki. In this approval, written informed consent was waived because of the anonymous and retrospective nature of this study.

### Gallbladder carcinoma cells, transfection, and western blot

The SNU-308 gallbladder carcinoma cell line was purchased from the Korean Cell Line Bank (KCLB, Seoul, Republic of Korea) and cultured in RPMI-1640 culture media with 10% fetal bovine serum (Gibco BRL, Gaithersburg, MD). SNU-308 cells were transfected with control shRNA, shRNA for FAM83H (GenePharma, Shanghai, China), empty vector, or a vector overexpressing FAM83H (Catalog #; EX-Y4473-M03, accession #; NM_198488, GeneCopoeia, Rockville, MD) by using Lipofectamine® 2000 DNA transfection reagent (Thermo Fisher Scientific, Waltham, MA). The FAM83H duplex had the sense and antisense sequences 5′-CACCGCTCATCTTCAGCACGTCACATTCAAGAGATGTGACGTGCTGAAGATGAGCTTTTTTG-3′ and 5′-GATCCAAAAAAGCTCATCTTCAGCACGTCACATCTCTTGAATGTGACGTGCTGAAGATGAGC-3′, respectively. The protein lysate from transfected cells was prepared via PRO-PREP Protein Extraction Solution (iNtRON Biotechnology, Seongnam, Korea) and blotted with antibodies for FAM83H (1:100, Bethyl Laboratories, Montgomery, TX), ZNF16 (1:250, Novus Biologicals, Centennial, CO), and actin (Santa Cruz Biotechnology, Santa Cruz, CA).

### Immunohistochemical staining and scoring

The expression of FAM83H and ZNF16 in gallbladder carcinomas were evaluated with immunohistochemical staining of tissue microarray sections. The core of the tissue microarray was obtained from the area composed primarily of tumor cells without degeneration. Two 3.0 mm cores per case were established in the tissue microarray. The tissue sections were deparaffinized and boiled for 20 min in a microwave oven in pH 6.0 antigen retrieval solution (DAKO, Glostrup, Denmark). Thereafter, the tissue sections were incubated with primary antibodies for FAM83H (1:100, Bethyl Laboratories, Montgomery, TX) and ZNF16 (1:250, Novus Biologicals, Centennial, CO). The immunohistochemical staining for FAM83H and ZNF16 was evaluated by two pathologists (KYJ and HSP) with consensus. The expressions of FAM83H and ZNF16 were separately evaluated according to their nuclear and cytoplasmic expression without clinicopathological information. Immunohistochemical staining was scored for the staining intensity (negative, score 0; weak, score 1; intermediate, score 2; strong, point 3) and staining area (no staining, score 0; ~ 1%, score 1; 2 ~ 10%, score 2; 11 ~ 33%, score 3; 34 ~ 66%, score 4; 67 ~ 100%, score 5) [6, 8, 20, 21]. The score for each tissue microarray core was obtained by adding the staining intensity score and the staining area score. Thereafter, the final immunohistochemical staining score was obtained by adding the scores from the two tissue microarray cores. Therefore, the immunohistochemical staining score ranged from zero to sixteen.

### Statistical analysis

The immunohistochemical expression of nuclear or cytoplasmic FAM83H and ZNF16 were classified into negative and positive subgroups. The cut-off points for the immunostaining scores were set by receiver operating characteristic curve analysis at the highest predictive point for the death of gallbladder carcinoma patients [8, 22]. The cut-off point has the highest area under the curve. The prognosis of gallbladder carcinoma patients was determined for overall survival (OS) and relapse-free survival (RFS) through December 2013. In OS analysis, an event was the death of the patient from gallbladder carcinoma and the duration calculated from the date of operation of gallbladder cancer to the date of last contact. The patients who died from other causes or were alive at last contact were treated as censored. An event in RFS analysis was a relapse of cancer or death of the patient from gallbladder carcinoma. The patients who died from other causes or were alive at last contact without relapse were treated as censored. The prognostic value was calculated with Cox proportional hazards regression analysis and the survival curve was generated with Kaplan-Meier survival analysis. The relationships among clinicopathological factors were determined via Pearson’s chi-square test. SPSS software (IBM, version 22.0, Armonk, NY) was used for statistical analysis with *p* values less than 0.05 being considered statistically significant.

## Results

### The association of clinicopathologic factors with the expressions of FAM83H and ZNF16 in gallbladder carcinomas

**Fig. 1:**
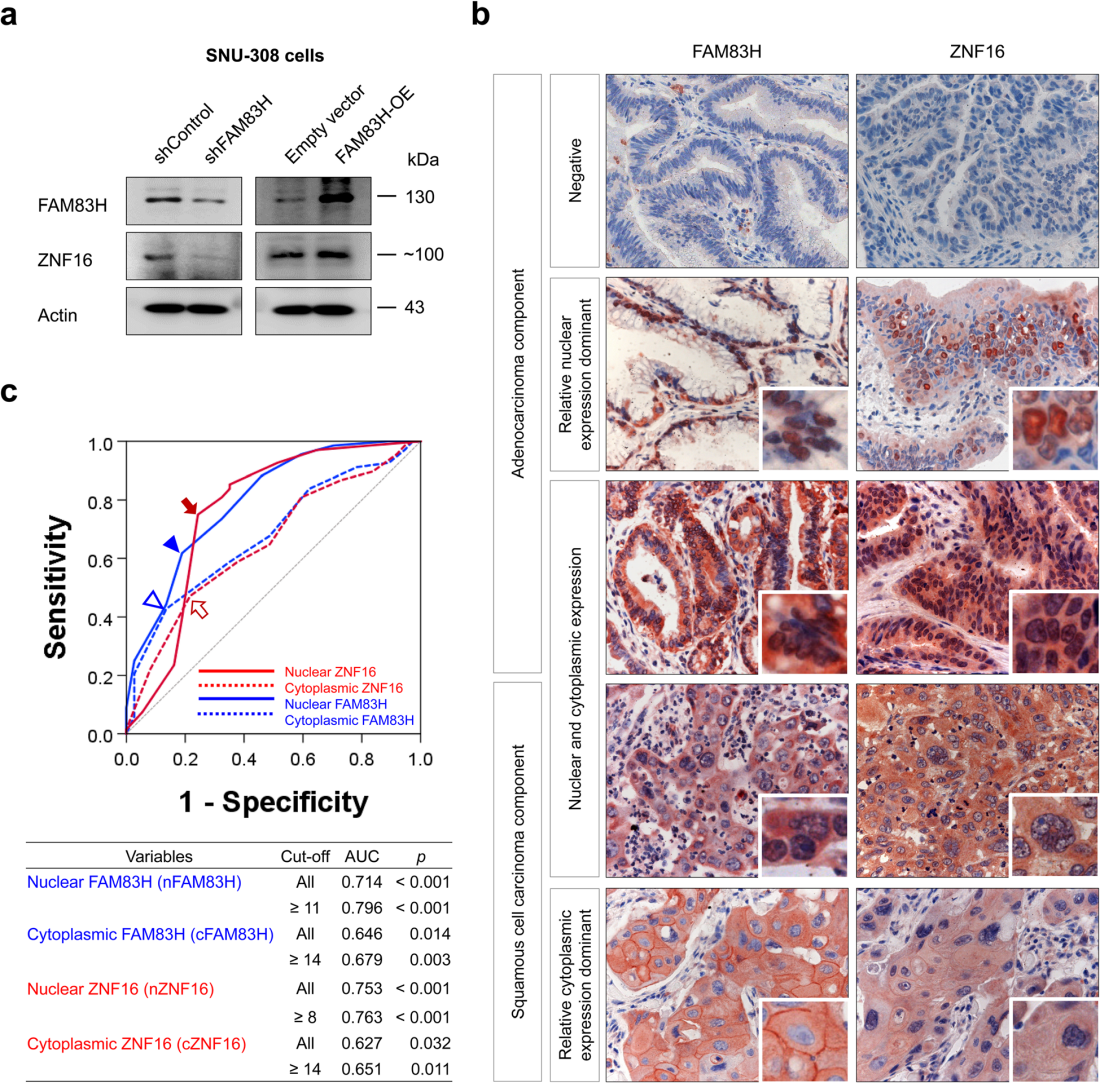
Western blot and immunohistochemical expression of FAM83H and ZNF16 and statistical analysis in gallbladder carcinomas. **a** SNU-308 gallbladder carcinoma cells were transfected with control shRNA, shRNA for FAM83H, empty vector, or a vector overexpressing FAM83H (FAM83H-OE) and performed western blot for FAM83H, ZNF16, and actin. **b** FAM83H and ZNF16 are expressed in both the cytoplasm and nuclei of cancer cells. Original magnification: × 400. **c** Receiver operating characteristic curve analysis to determine cut-off points for the expression of nuclear FAM83H (blue arrowhead), cytoplasmic FAM83H (empty blue arrowhead), nuclear ZNF16 (red arrow), and cytoplasmic ZNF16 (empty red arrow). The cut-off points indicate the point of the highest area under the curve (AUC) to predict the death of gallbladder carcinoma patients

To verify the specificity of the antibodies for FAM83H and ZNF16, western blots for FAM83H and ZNF16 were performed on SNU-308 gallbladder carcinoma cells after inducing knock-down or overexpression of FAM83H. Knock-down of FAM83H decreased expression of FAM83H and ZNF16, and overexpression of FAM83H increased expression of FAM83H and ZNF16 (Fig. 1a). Immunohistochemical expression of FAM83H and ZNF16 in gallbladder carcinomas are presented in Fig. 1b. The expression of FAM83H and ZNF16 were detected in both cytoplasmic and nuclear areas of adenocarcinoma components (Fig. 1b). In squamous cell carcinoma components, the expression of FAM83H and ZNF16 were detected in the cytoplasmic membrane, cytoplasm, and nuclei of tumor cells (Fig. 1b). Immunohistochemical expression of FAM83H and ZNF16 were grouped into negative or positive groups by receiver operating characteristic curve analysis. The cut-off points for the expression of nuclear FAM83H, cytoplasmic FAM83H, nuclear ZNF16, and cytoplasmic ZNF16 were eleven, fourteen, eight, and fourteen, respectively (Fig. 1c). With these cut-off values, nuclear FAM83H expression was significantly associated with histologic grade (*p* = 0.044) and the expression of cytoplasmic FAM83H (*p* <  0.001) and nuclear ZNF16 (*p* = 0.002) (Table 1). Cytoplasmic expression of FAM83H was significantly associated with distant metastasis (*p* = 0.022), tumor stage (*p* = 0.040), histologic grade (*p* = 0.003), and the expression of nuclear ZNF16 (*p* = 0.019) and cytoplasmic ZNF16 (*p* = 0.009) (Table 1). Positivity for nuclear ZNF16 was significantly associated with tumor stage (*p* = 0.028), T category of the tumor stage (*p* = 0.014), histologic grade (*p* <  0.001), and the cytoplasmic expression of ZNF16 (*p* <  0.001) (Table 1). Cytoplasmic expression of ZNF16 was clearly associated with the age of the patients (*p* = 0.046), tumor stage (*p* = 0.003), and T category of the tumor stage (*p* <  0.001) (Table 1).

**Table 1:** Clinicopathologic variables and the expression of FAM83H and ZNF16 in gallbladder carcinomas

Characteristics	No.	nuclear FAM83H		cytoplasmic FAM83H		nuclear ZNF16		cytoplasmic ZNF16	
		Positive	*p*	Positive	*p*	Positive	*p*	Positive	*p*
Age, years									
< 65	55	23 (42%)	0.296	15 (27%)	0.241	30 (55%)	0.573	16 (29%)	0.046
≥ 65	50	26 (52%)		19 (38%)		30 (60%)		24 (48%)	
Sex									
Male	55	26 (47%)	0.896	19 (35%)	0.619	31 (56%)	0.866	22 (40%)	0.673
Female	50	23 (46%)		15 (30%)		29 (58%)		18 (36%)	
CEA									
Normal	86	39 (45%)	0.565	27 (31%)	0.646	50 (58%)	0.661	35 (41%)	0.243
Elevated	19	10 (53%)		7 (37%)		10 (53%)		5 (26%)	
CA19–9									
Normal	71	30 (42%)	0.190	23 (32%)	0.997	36 (51%)	0.054	25 (35%)	0.379
Elevated	34	19 (56%)		11 (32%)		24 (71%)		15 (44%)	
TNM stage									
I	25	7 (28%)	0.122	5 (20%)	0.040	8 (32%)	0.028	3 (12%)	0.003
II	37	19 (51%)		11 (30%)		23 (62%)		20 (54%)	
III	31	15 (48%)		10 (32%)		20 (65%)		10 (32%)	
IV	12	8 (67%)		8 (67%)		9 (75%)		7 (58%)	
T category									
T1	26	7 (27%)	0.101	5 (19%)	0.071	8 (31%)	0.014	3 (12%)	< 0.001
T2	50	26 (52%)		15 (30%)		31 (62%)		27 (54%)	
T3	25	13 (52%)		11 (44%)		18 (72%)		7 (28%)	
T4	4	3 (75%)		3 (75%)		4 (100%)		3 (75%)	
LN metastasis									
Absence	79	34 (43%)	0.194	25 (32%)	0.779	43 (54%)	0.328	32 (41%)	0.375
Presence	26	15 (58%)		9 (35%)		17 (65%)		8 (31%)	
Distant metastasis									
Absence	98	45 (46%)	0.565	29 (30%)	0.022	54 (55%)	0.114	36 (37%)	0.283
Presence	7	4 (57%)		5 (71%)		6 (86%)		4 (57%)	
Lymphovascular invasion									
Absence	95	44 (46%)	0.824	29 (31%)	0.211	52 (55%)	0.125	38 (40%)	0.215
Presence	10	5 (50%)		5 (50%)		8 (80%)		2 (20%)	
Histologic type									
Adenocarcinoma NOS	101	48 (48%)	0.571	32 (32%)	0.348	58 (57%)	0.485	38 (38%)	0.435
Adenosquamous carcinoma	3	1 (33%)		2 (67%)		2 (67%)		2 (67%)	
Squamous cell carcinoma NOS	1	0 (0%)		0 (0%)		0 (0%)		0 (0%)	
Histologic grade									
Low	43	15 (35%)	0.044	7 (16%)	0.003	15 (35%)	< 0.001	12 (28%)	0.073
High	62	34 (55%)		27 (44%)		45 (73%)		28 (45%)	
cytoplasmic ZNF16									
Negative	65	26 (40%)	0.081	25 (23%)	0.009	28 (43%)	< 0.001		
Positive	40	23 (58%)		19 (48%)		32 (80%)			
nuclear ZNF16									
Negative	45	13 (29%)	0.002	9 (20%)	0.019				
Positive	60	36 (60%)		25 (42%)					
cytoplasmic FAM83H									
Negative	71	24 (34%)	< 0.001						
Positive	34	25 (74%)							

*Abbreviations: CEA* carcinoembryonic antigen, *CA19–9* carbohydrate antigen 19–9, *LN* lymph node, *NOS* not otherwise specified

### The expressions of FAM83H and ZNF16 are associated with shorter survival of gallbladder carcinoma patients

**Fig. 2:**
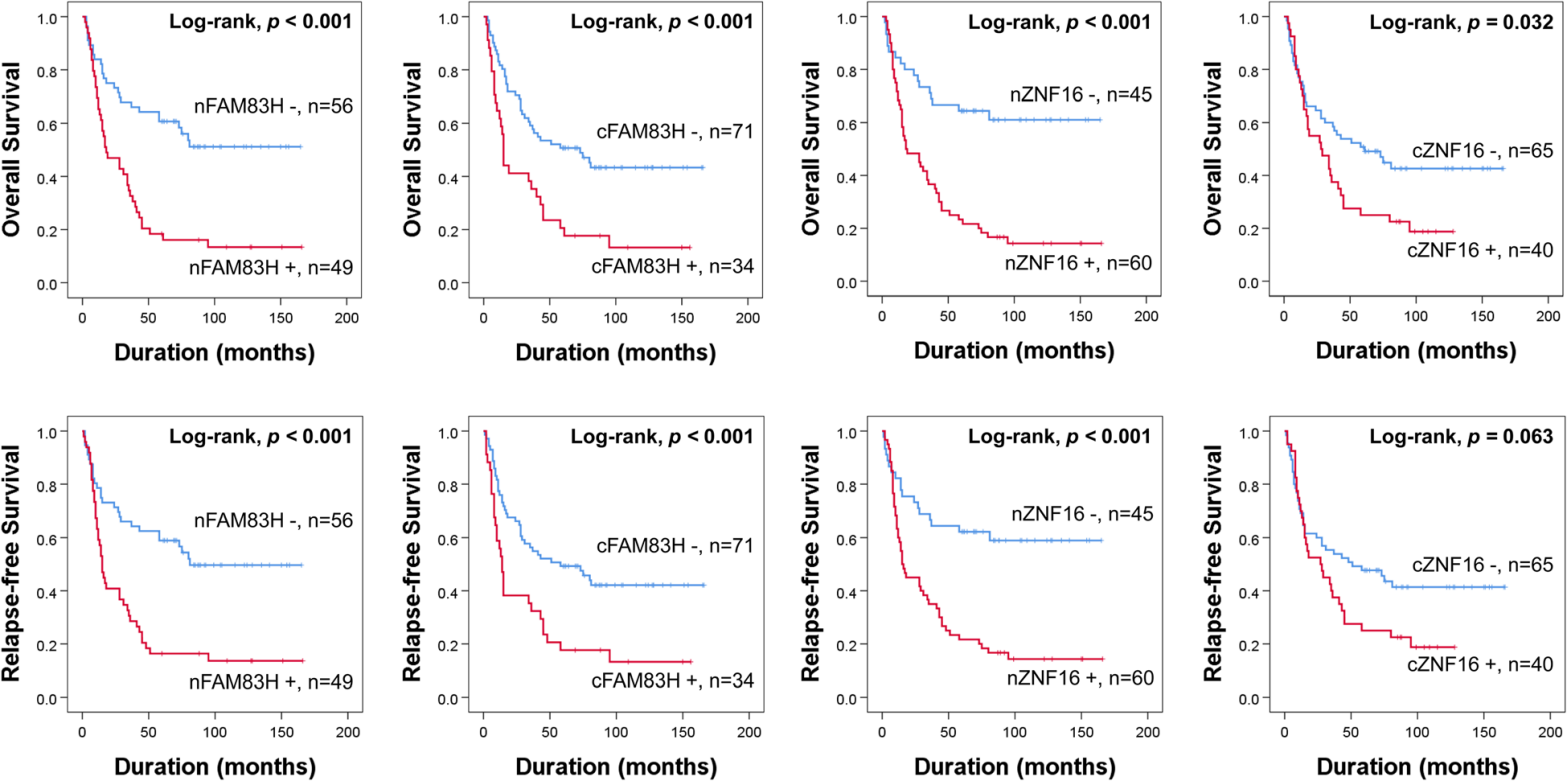
Survival analysis according to the expression of FAM83H and ZNF16 in gallbladder carcinomas. Kaplan-Meier survival curves for overall survival and relapse-free survival of gallbladder carcinoma patients according to the expression of nuclear FAM83H (nFAM83H), cytoplasmic FAM83H (cFAM83H), nuclear ZNF16 (nZNF16), and cytoplasmic ZNF16 (cZNF16)

The factors clearly associated with OS or RFS of gallbladder carcinomas were age, preoperative serum level of CA19–9, tumor stage, T category of tumor stage, lymph node metastasis, distant metastasis, lymphovascular invasion, histologic type, histologic grade, and the expression of nuclear FAM83H (OS; *p* <  0.001, RFS; *p* <  0.001), cytoplasmic FAM83H (OS; *p* <  0.001, RFS; *p* = 0.001), nuclear ZNF16 (OS; *p* <  0.001, RFS; *p* <  0.001), and cytoplasmic ZNF16 (OS; *p* = 0.035, RFS; *p* = 0.068) (Table 2). Nuclear FAM83H positivity predicted a 2.823-fold [95% confidence interval (95% CI); 1.716–4.646] greater risk of death and a 2.685-fold (95% CI; 1.640–4.395) greater risk of relapse or death of gallbladder carcinoma patients. Cytoplasmic FAM83H positivity predicted a 2.292-fold (95% CI; 1.413–3.720) greater risk of death and a 2.201-fold (95% CI; 1.360–3.564) greater risk of relapse or death of gallbladder carcinoma patients. Patients with nuclear ZNF16 positive carcinomas had a 3.287-fold (95% CI; 1.888–5.722) greater risk of death and a 3.038-fold (95% CI; 1.765–5.229) greater risk of relapse or death from gallbladder carcinoma. Patients with cytoplasmic ZNF16 positive carcinomas had a 1.675-fold (95% CI; 1.038–2.703) greater risk of death from gallbladder carcinoma (Table 3). Figure 2 presents Kaplan-Meier survival curves for OS and RFS of gallbladder carcinoma patients according to the nuclear and cytoplasmic expression of FAM83H and ZNF16.

**Table 2:** Univariate Cox proportional hazards regression analysis for overall survival and relapse-free survival in gallbladder carcinoma patients

Characteristics	No.	OS	*p*	RFS	*p*
		HR (95% CI)		HR (95% CI)	
Age, y ≥ 65 (vs. < 65)	50/105	2.720 (1.655–4.470)	< 0.001	2.600 (1.592–4.245)	< 0.001
Sex, female (vs. male)	50/105	0.853 (0.529–1.376)	0.516	0.796 (0.495–1.280)	0.347
CEA, elevated (vs. normal)	19/105	1.284 (0.701–2.352)	0.419	1.194 (0.653–2.185)	0.564
CA19–9, elevated (vs. normal)	34/105	1.864 (1.143–3.040)	0.013	1.809 (1.112–2.942)	0.017
TNM stage					
I	25/105	1	< 0.001	1	< 0.001
II	37/105	2.459 (1.094–5.529)	0.030	2.619 (1.170–5.862)	0.019
III	31/105	5.495 (2.466–12.247)	< 0.001	5.129 (2.300–11.436)	< 0.001
IV	12/105	18.262 (6.941–48.048)	< 0.001	16.700 (6.374–43.750)	< 0.001
T category					
T1	26/105	1	< 0.001	1	< 0.001
T2	50/105	2.954 (1.358–6.425)	0.006	3.051 (1.406–6.622)	0.005
T3	25/105	10.560 (4.641–24.030)	< 0.001	9.672 (4.252–22.004)	< 0.001
T4	4/105	11.670 (3.379–40.309)	< 0.001	12.256 (3.542–42.413)	< 0.001
LN metastasis, presence (vs. absence)	26/105	2.019 (1.207–3.376)	0.007	1.898 (1.137–3.168)	0.014
Distant metastasis, presence (vs. absence)	7/105	7.082 (2.971–16.885)	< 0.001	5.419 (2.339–12.555)	< 0.001
Lymphovascular invasion, presence (vs. absence)	10/105	3.166 (1.551–6.465)	0.002	2.886 (1.416–5.881)	0.004
Histologic type					
adenocarcinoma NOS	101/105	1	0.003	1	0.005
adenosquamous carcinoma	3/105	4.268 (1.320–13.800)	0.015	3.587 (1.113–11.563)	0.032
squamous cell carcinoma NOS	1/105	15.503 (1.903–126.302)	0.010	15.437 (1.894–125.789)	0.011
Histologic grade, high (vs. low)	62/105	3.197 (1.855–5.510)	< 0.001	2.989 (1.753–5.097)	< 0.001
cytoplasmic ZNF16, positive (vs. negative)	40/105	1.675 (1.038–2.703)	0.035	1.557 (0.968–2.505)	0.068
nuclear ZNF16, positive (vs. negative)	60/105	3.287 (1.888–5.722)	< 0.001	3.038 (1.765–5.229)	< 0.001
cytoplasmic FAM83H, positive (vs. negative)	34/105	2.292 (1.413–3.720)	< 0.001	2.201 (1.360–3.564)	0.001
nuclear FAM83H, positive (vs. negative)	49/105	2.823 (1.716–4.646)	< 0.001	2.685 (1.640–4.395)	< 0.001

*Abbreviations: OS* overall survival, *RFS* relapse-free survival, *HR* hazard ratio, *95% CI* 95% confidence interval, *CEA* carcinoembryonic antigen, *CA19–9* carbohydrate antigen 19–9, *LN* lymph node, *NOS* not otherwise specified

**Table 3:** Multivariate Cox regression analysis for overall survival and relapse-free survival

Characteristics	OS	*p*	RFS	*p*
	HR (95% CI)		HR (95% CI)	
Age, y ≥ 65 (vs. < 65)	3.698 (2.194–6.233)	< 0.001	3.484 (2.085–5.824)	< 0.001
TNM stage				
I	1	< 0.001	1	< 0.001
II	2.304 (1.021–5.197)	0.044	2.473 (1.101–5.556)	0.028
III	6.168 (2.715–14.011)	< 0.001	5.819 (2.554–13.259)	< 0.001
IV	25.342 (8.985–71.472)	< 0.001	23.060 (8.258–64.396)	< 0.001
Nuclear FAM83H, positive (vs. negative)	2.094 (1.243–3.525)	0.005	2.108 (1.260–3.527)	0.005

*Abbreviations: OS* overall survival, *RFS* relapse-free survival, *HR* hazard ratio, *95% CI* 95% confidence interval. Variables considered in the multivariate analysis were age, preoperative serum level of CA19–9, tumor stage, T category of tumor stage, lymph node metastasis, distant metastasis, lymphovascular invasion, histologic type, histologic grade, and the expression of nuclear FAM83H, cytoplasmic FAM83H, nuclear ZNF16, and cytoplasmic ZNF16

Multivariate analysis was performed with the factors clearly associated with OS or RFS; age, preoperative serum level of CA19–9, tumor stage, T category of tumor stage, lymph node metastasis, distant metastasis, lymphovascular invasion, histologic type, histologic grade, and the expression of nuclear FAM83H, cytoplasmic FAM83H, nuclear ZNF16, and cytoplasmic ZNF16. Multivariate analysis showed age (OS; *p* <  0.001, RFS; *p* <  0.001), tumor stage (OS; overall *p* <  0.001, RFS; overall *p* <  0.001), and nuclear FAM83H expression (OS; *p* = 0.005, RFS; *p* = 0.005) to be independent prognostic indicators of gallbladder carcinoma patients (Table 3). Nuclear FAM83H positivity predicted a 2.094-fold (95% CI; 1.243–3.525) greater risk of death and a 2.108-fold (95% CI; 1.260–3.527) greater risk of relapse or death of gallbladder carcinoma patients by the multivariate analysis (Table 3).

### The co-expression pattern of nuclear FAM83H and nuclear ZNF16 was associated with shorter survival of gallbladder carcinoma patients

**Fig. 3:**
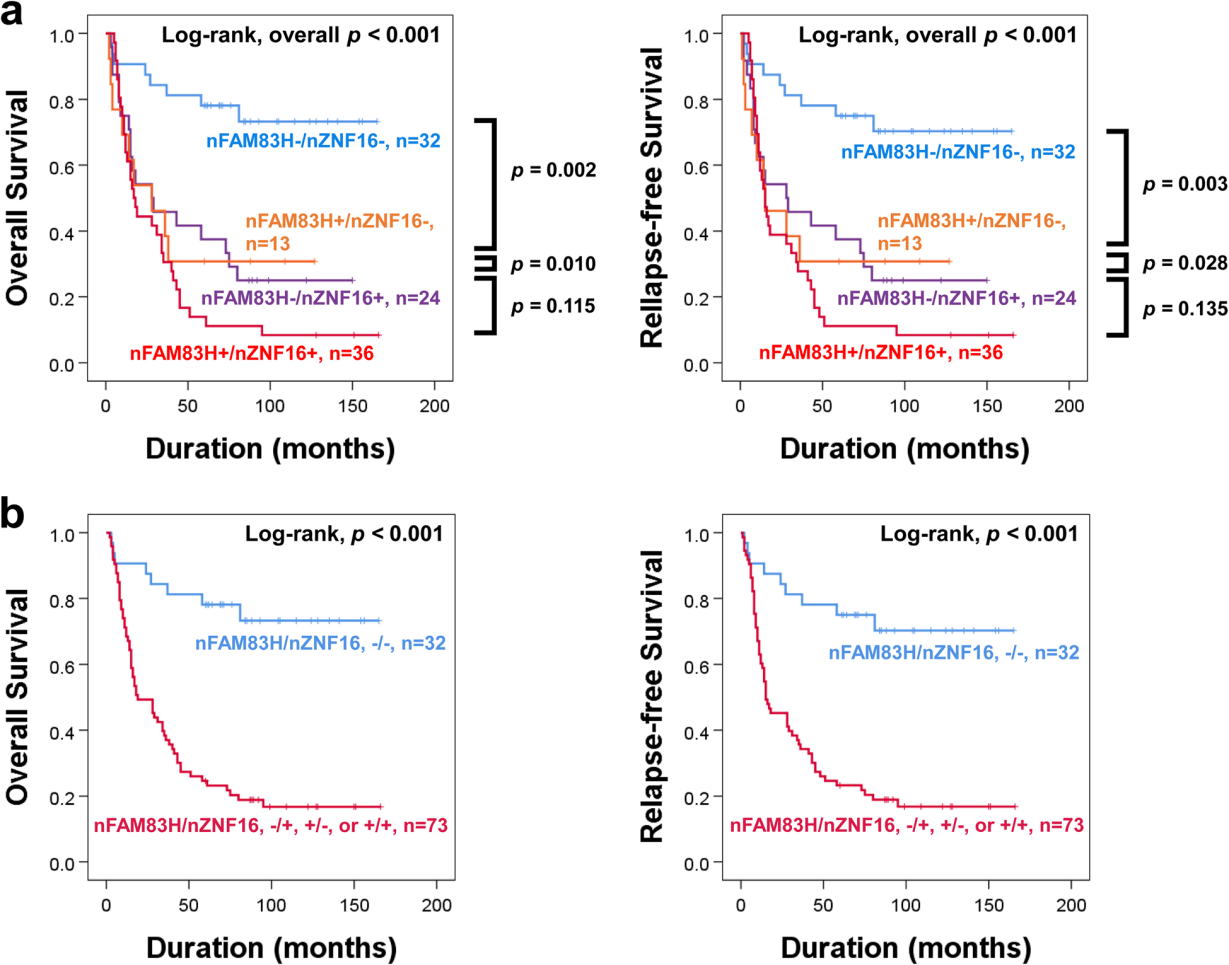
Survival analysis according to co-expression patterns of nuclear FAM83H and nuclear ZNF16 in gallbladder carcinoma patients. **a** Survival analysis for overall survival and relapse-free survival in four subgroups of gallbladder carcinoma according to the expression of nuclear FAM83H (nFAM83H) and nuclear ZNF16 (nZNF16): nFAM83H^−^/nZNF16^−^, nFAM83H^−^/nZNF16^+^, nFAM83H^+^/nZNF16^−^, and nFAM83H^+^/nZNF16^+^ subgroups. **b** Survival analysis for overall survival and relapse-free survival in two groups of gallbladder carcinomas: [nFAM83H^−^/nZNF16^−^] and [nFAM83H^−^/nZNF16^+^, nFAM83H^+^/nZNF16^−^ or nFAM83H^+^/nZNF16^+^] subgroups

In our results, the expression of FAM83H was significantly associated with ZNF16 expression. Moreover, the expression of nuclear FAM83H, cytoplasmic FAM83H, nuclear ZNF16, and cytoplasmic ZNF16 were associated with the survival of gallbladder carcinoma patients. In addition, as shown in Table 2, the prognostic predictability of nuclear expressions of FAM83H and ZNF16 were stronger than cytoplasmic expressions of FAM83H and ZNF16 when incorporating their hazard ratios and *P* values. Therefore, based on the relationship between FAM83H and ZNF16 expression and the prognostic value of the nuclear expression of FAM83H and ZNF16, we further evaluated the clinical significance of the co-expression pattern of nuclear FAM83H and nuclear ZNF16 (nFAM83H/nZNF16). When we sub-grouped gallbladder carcinomas into nFAM83H^−^/nZNF16^−^, nFAM83H^−^/nZNF16^+^, nFAM83H^+^/nZNF16^−^, and nFAM83H^+^/nZNF16^+^ subgroups, the nFAM83H^−^/nZNF16^−^ subgroup had the longest survival and the nFAM83H^+^/nZNF16^+^ subgroup had the shortest survival (Fig. 3a) (Table 4). However, the difference in survival among nFAM83H^−^/nZNF16^+^, nFAM83H^+^/nZNF16^−^, and nFAM83H^+^/nZNF16^+^ subgroups were minimal (Fig. 3a). Based on these survival analyses, we grouped gallbladder carcinomas into two subgroups: favorable (nFAM83H^−^/nZNF16^−^) and poor (nFAM83H^−^/nZNF16^+^, nFAM83H^+^/nZNF16^−^, or nFAM83H^+^/nZNF16^+^) subgroups (Fig. 3b) (Table 4). These prognostic subgroups were significantly associated with tumor stage (*p* = 0.016), T category of tumor stage (*p* = 0.006), and histologic grade (*p* <  0.001) (Table 5). In univariate Cox regression analysis, the poor prognostic subgroup with co-expression of nFAM83H/nZNF16 predicted a 5.463-fold (95% CI; 2.598–11.487, *p* <  0.001) higher risk of death and a 4.796-fold (95% CI; 2.367–9.717, *p* <  0.001) higher risk of relapse or death of patients (Table 6). In multivariate analysis, the co-expression of nFAM83H/nZNF16 was also an independent indicator of poor prognosis of gallbladder carcinoma patients (Table 6). The poor prognostic subgroup co-expressing nFAM83H/nZNF16 had a 4.808-fold (95% CI; 2.143–10.791, *p* <  0.001) higher risk of death and a 4.204-fold (95% CI; 1.958–9.029, *p* <  0.001 higher risk of relapse or death of patients compared with the favorable prognostic subgroup (Table 6).

**Table 4:** Five- and ten-year overall survival and relapse-free survival according to co-expression patterns of nuclear FAM83H and nuclear ZNF16

Co-expression pattern of nFAM83H and nZNF16	No.	5y-OS (%)	10y-OS (%)	5y-RFS (%)	10y-RFS (%)
Co-expression Model 1					
nFAM83H−/nZNF16-	32	78	73	75	70
nFAM83H−/nZNF16+	24	38	25	38	25
nFAM83H+/nZNF16-	13	31	31	31	31
nFAM83H+/nZNF16+	36	14	8	11	8
Co-expression Model 2					
nFAM83H−/nZNF16-	32	78	73	75	70
nFAM83H−/nZNF16+, nFAM83H+/nZNF16-, or nFAM83H+/nZNF16+	73	25	17	23	17

*Abbreviations: nFAM83H* nuclear expression of FAM83H, *nZNF16* nuclear expression of ZNF16, *5y-OS* overall survival rate at 5 years, *10y-OS* overall survival rate at 10 years, *5y-RFS*; relapse-free survival rate at 5 years, *10y-RFS* relapse-free survival rate at 10 years

**Table 5:** Clinicopathologic variables and co-expression patterns of nuclear FAM83H and nuclear ZNF16 in gallbladder carcinomas

Characteristics	No.	nFAM83H/nZNF16 co-expression		
		−/−	−/+, +/−, or +/+	*p*
Age, years				
< 65	55	20 (36%)	35 (64%)	0.169
≥ 65	50	12 (24%)	38 (76%)	
Sex				
Male	55	16 (29%)	39 (71%)	0.746
Female	50	16 (32%)	34 (68%)	
CEA				
Normal	86	25 (29%)	61 (71%)	0.505
Elevated	19	7 (37%)	12 (63%)	
CA19–9				
Normal	71	25 (35%)	46 (65%)	0.128
Elevated	34	7 (21%)	27 (79%)	
TNM stage				
I	25	14 (56%)	11 (44%)	0.016
II	37	9 (24%)	28 (76%)	
III	31	7 (23%)	24 (77%)	
IV	12	2 (17%)	10 (83%)	
T category				
T1	26	15 (58%)	11 (42%)	0.006
T2	50	12 (24%)	38 (76%)	
T3	25	4 (16%)	21 (84%)	
T4	4	1 (25%)	3 (75%)	
LN metastasis				
Absence	79	27 (34%)	52 (66%)	0.151
Presence	26	5 (19%)	21 (81%)	
Distant metastasis				
Absence	98	31 (32%)	67 (68%)	0.335
Presence	7	1 (14%)	6 (86%)	
Lymphovascular invasion				
Absence	95	31 (33%)	64 (67%)	0.139
Presence	10	1 (10%)	9 (90%)	
Histologic type				
Adenocarcinoma NOS	101	31 (31%)	70 (69%)	0.165
Adenosquamous carcinoma	3	0 (0%)	3 (100%)	
Squamous cell carcinoma NOS	1	1 (100%)	0 (0%)	
Histologic grade				
Low	43	21 (49%)	22 (51%)	< 0.001
High	62	11 (18%)	51 (82%)	

*Abbreviations: nFAM83H* nuclear expression of FAM83H, *nZNF16* nuclear expression of ZNF16, *CEA* carcinoembryonic antigen, *CA19–9* carbohydrate antigen 19–9, *LN* lymph node, *NOS* not otherwise specified

**Table 6:** Univariate and multivariate Cox regression analysis for overall survival and relapse-free survival according to the co-expression patterns of nuclear FAM83H and nuclear ZNF16 in gallbladder carcinomas

Characteristics	OS	*p*	RFS	*p*
	HR (95% CI)		HR (95% CI)	
Univariate analysis				
nFAM83H/nZNF16, +/−, −/+, or +/+ (vs. −/−)	5.463 (2.598–11.487)	< 0.001	4.796 (2.367–9.717)	< 0.001
Multivariate analysis				
Age, y ≥ 65 (vs. < 65)	3.383 (1.989–5.755)	< 0.001	3.249 (1.925–5.484)	< 0.001
TNM stage				
I	1	< 0.001	1	< 0.001
II	1.897 (0.830–4.257)	0.130	2.019 (0.895–4.558)	0.091
III	5.318 (2.307–12.257)	< 0.001	4.939 (2.219–11.459)	< 0.001
IV	25.409 (8.833–73.088)	< 0.001	22.922 (1.988–65.772)	< 0.001
Histologic type				
adenocarcinoma NOS	1	0.008	1	0.010
adenosquamous carcinoma	0.726 (0.214–2.458)	0.607	0.644 (0.190–2.180)	0.480
squamous cell carcinoma NOS	35.714 (3.628–351.524)	0.002	29.708 (3.079–286.603)	0.003
nFAM83H/nZNF16, +/−, −/+, or +/+ (vs. −/−)	4.808 (2.143–10.791)	< 0.001	4.204 (1.958–9.029)	< 0.001

*Abbreviations: OS* overall survival, *RFS* relapse-free survival, *HR* hazard ratio, *95% CI* 95% confidence interval; nFAM83H/nZNF16, co-expression patterns of nuclear FAM83H, and nuclear ZNF16. Variables considered in the multivariate analysis were age, preoperative serum level of CA19–9, tumor stage, T category of tumor stage, lymph node metastasis, distant metastasis, lymphovascular invasion, histologic type, histologic grade, and the co-expression patterns of nuclear FAM83H and nuclear ZNF16

**Fig. 4:**
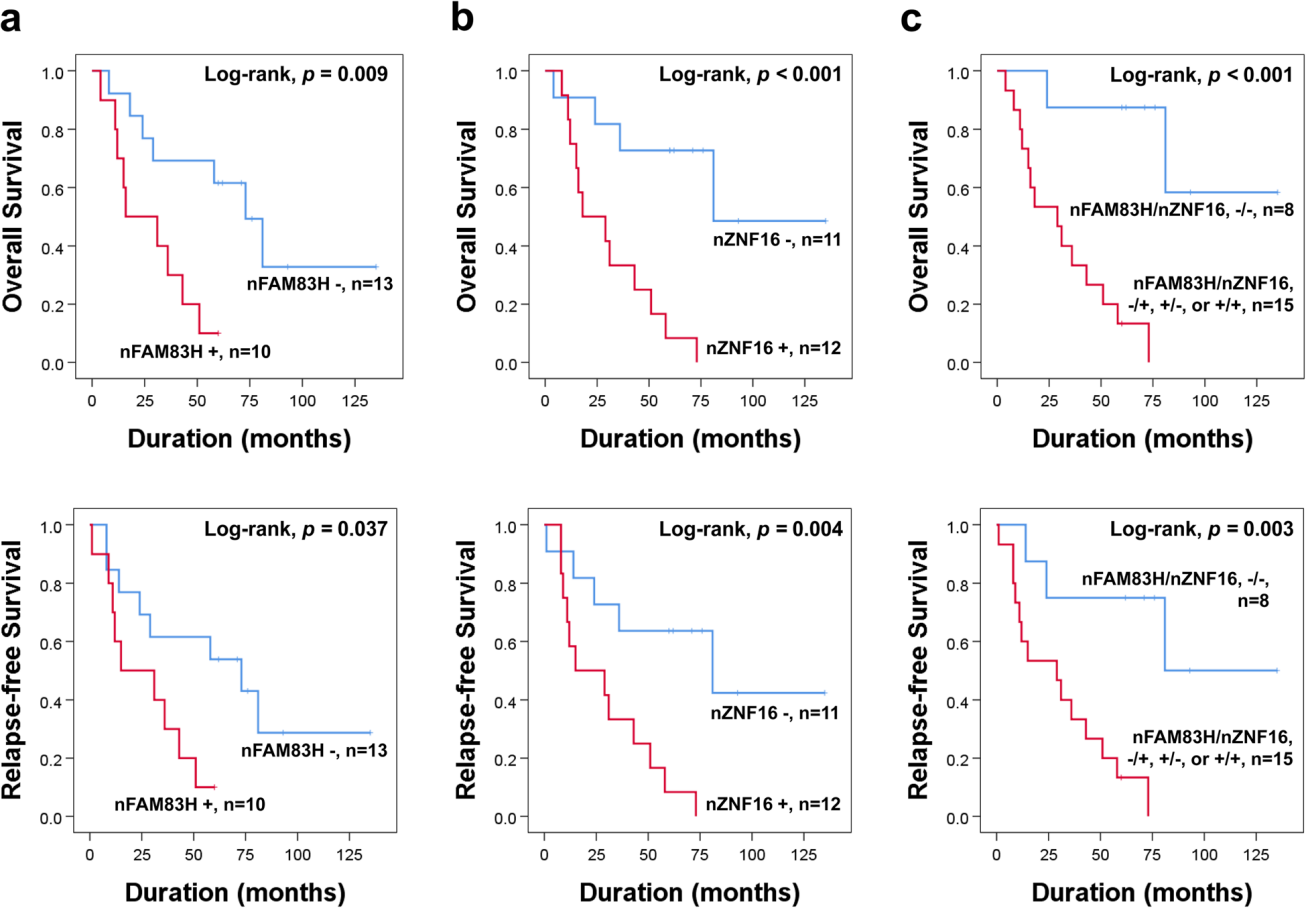
Survival analysis according to the individual and co-expression patterns of nuclear FAM83H and nuclear ZNF16 in 23 gallbladder carcinoma patients who received adjuvant chemotherapy. **a** Survival analysis for overall survival and relapse-free survival according to the expression of nuclear FAM83H (nFAM83H). **b** Survival analysis for overall survival and relapse-free survival according to expression of nuclear ZNF16 (nZNF16). **c** Survival analysis in two prognostic groups of gallbladder carcinomas according to co-expression patterns of nuclear FAM83H and nuclear ZNF16: [nFAM83H^−^/nZNF16^−^] and [nFAM83H^−^/nZNF16^+^, nFAM83H^+^/nZNF16^−^ or nFAM83H^+^/nZNF16^+^] subgroups

Furthermore, we evaluated the prognostic significance of the individual and co-expression patterns of nuclear FAM83H and nuclear ZNF16 in 23 gallbladder carcinoma patients who received adjuvant chemotherapy. As shown in Fig. 4, individual expression of nuclear FAM83H and nuclear ZNF16, and two prognostic subgroups according to the co-expression patterns of nFAM83H/nZNF16 [(nFAM83H^−^/nZNF16^−^) versus (nFAM83H^−^/nZNF16^+^, nFAM83H^+^/nZNF16^−^, or nFAM83H^+^/nZNF16^+^) subgroups] were significantly associated with OS and RFS (Fig. 4).

## Discussion

In this study, we demonstrate a positive correlation between immunohistochemical expression of FAM83H and ZNF16 in gallbladder cancers. There was also a close association between the expression of FAM83H and ZNF16 and advanced clinicopathological factors. Moreover, the positivity for the expression of nuclear FAM83H, cytoplasmic FAM83H, nuclear ZNF16, and cytoplasmic ZNF16 were significantly associated with shorter survival of gallbladder carcinoma patients. Especially, nuclear FAM83H expression was an independent marker of poor prognosis of gallbladder carcinoma patients. Concerning the subcellular localization of FAM83H, FAM83H was present in the cytoplasmic membrane and the cytosol [3, 4, 23]. Subsequently, it was shown that the nuclear expression of FAM83H is present in some cases of colon carcinomas, and its nuclear localization has been suggested be involved in tumorigenesis [24]. Furthermore, in hepatocellular carcinoma and clear cell renal cell carcinoma, nuclear FAM83H expression was an independent prognostic indicator of cancer patients [5, 8]. In addition, FAM83H had cooperative roles with MYC and Wnt/β-catenin pathways in cancer progression [5, 6]. MYC transcriptionally controlled the expression of FAM83H and FAM83H is involved in the stabilization of β-catenin and consequent transcriptional activation of the canonical Wnt pathway. Therefore, when considering the roles of the nuclear localization of MYC and β-catenin, nuclear FAM83H also has a role in the nuclei of cells in association with MYC/β-catenin. In addition, FAM83H localized to nuclear speckles and interacted with SON, a protein in nuclear speckle, and FAM83H is involved in nuclear recruitment of casein kinase 1α in colorectal cancer cells [24]. Therefore, it has been suggested that FAM83H might be involved in cancer progression via extensive interaction with nuclear proteins. However, in contrast, cytoplasmic FAM83H expression was more predictive than nuclear FAM83H expression for the survival of osteosarcoma patients [6]. Therefore, although the prognostic significance of FAM83H expression varied according to its subcellular localization with different cancer type, it has been suggested that the overall expression of FAM83H in the cell could be vital in the progression of cancers because knock-down of FAM83H suppressed cancer progression and overexpression of FAM83H stimulated cancer progression [5, 6]. However, further study is needed to clarify the mechanism and role of nuclear localization of FAM83H.

With regards to the subcellular localization of ZNF16, our results showed ZNF16 expression in both the cytoplasm and nuclei of tumor cells. Furthermore, the nuclear expression of ZNF16 was indicated to be a potential prognostic factor of gallbladder carcinoma patients. Supportively, it has been reported that ZNF16 is expressed in both the cytoplasm and nucleus and nuclear ZNF16 has an important role in the differentiation of cells [11, 25]. In addition, ZNF16 inhibited apoptosis and stimulated cell cycle progression by inhibiting INCA1 in K562 leukemia cells [26]. Therefore, when considering our finding that there is a significant association between nuclear ZNF16-positivity and higher tumor stage and histologic grade, nuclear expression of ZNF16 might be significantly involved in the progression of gallbladder carcinomas. However, the reports concerning the role of ZNF16 in human cancers, especially with regards to the prognostic significance of ZNF16 expression in human cancers, is limited. Therefore, we have searched the public database. In a search of the GEPIA database (Accessed 2 March 2020) [14], the mRNA expression of ZNF16 was elevated in breast cancer, cholangiocarcinoma, esophageal carcinoma, colon adenocarcinoma, head and neck squamous cell carcinoma, prostatic cancer and lung carcinoma compared with their normal counterpart tissues. In addition, although there is no data for gallbladder cancers, The Human Protein Atlas database (https://www.proteinatlas.org. Accessed 2 March 2020) [27] indicates that higher expression of ZNF16 mRNA is an indicator of poor prognosis of liver cancer (Log-rank, *p* = 0.008) and breast cancer (Log-rank, *p* = 0.041). However, higher expression of ZNF16 mRNA was associated with favorable prognosis of head and neck cancer (Log-rank, *p* <  0.001) and gastric cancer (Log-rank, *p* = 0.017) [27]. Therefore, further study is needed to clarify the role of ZNF16 in the progression of human cancers.

When considering the prognostic significance of the expression of FAM83H and ZNF16 in gallbladder carcinoma, it was expected that the expression of FAM83H and ZNF16 would be associated with standard prognostic variables. However, despite the significant correlation between cFAM83H/nZNF16/cZNF16 expression and tumor stage, there was no significant correlation between cFAM83H/nZNF16/cZNF16 expression and lymph node and distant metastasis. However, although not statistically significant, there is a tendency towards more positivity for FAM83/ZNF16 expression in patients with lymph node metastasis or distant metastasis. This might be related to the relatively low number of cases that have lymph node metastasis or distant metastasis. Therefore, further study is needed to clarify the relationship between FAM83H/ZNF16 expression and cancer progression. In addition, in the univariate analysis, the conventional prognostic indicators such as age of patient, serum level of CA19–9, tumor stage and TNM categories, lymphovascular invasion, histologic type, and histologic grade were significantly associated with OS and RFS of gallbladder carcinoma patients. In multivariate analysis, older age of patient and tumor stage were independent indicators of poor prognosis of gallbladder carcinoma patients. The reason why T, N, and M categories do not present as independent prognostic factors in the multivariate analysis might be related to the fact that T, N, and M categories are components of the TNM staging system. Therefore, our results support that the TNM staging system is important in the prediction of the survival of cancer patients.

Another interesting finding of our study is that the expression of FAM83H and ZNF16 were closely associated with each other in gallbladder cancers. In SNU-308 gallbladder carcinoma cells, knock-down of FAM83H decreased ZNF16 expression and overexpression of FAM83H increased expression of ZNF16. Furthermore, the co-expression pattern of nFAM83H/nZNF16 was a strong prognostic indicator of gallbladder carcinoma patients. Although no reports have investigated the relationship between FAM83H and ZNF16 in human cancers, we found a significant association between FAM83H and ZNF16 expression in human cancers in our search of the public database. Although there is no data for gallbladder cancers, a significant correlation between the expression of FAM83H and ZNF16 was seen in hepatobiliary cancers. The cBioPortal database (Accessed March 2, 2020) [12, 13] indicates that there is a significant correlation between the expression of mRNA of FAM83H and ZNF16 in cholangiocarcinoma (Spearman’s correlation, *R* = 0.70, *p* <  0.001). The GEPIA database (Accessed March 2, 2020) [14] showed a significant correlation between the expression of mRNA of FAM83H and ZNF16 in cholangiocarcinoma (Spearman’s correlation, *R* = 0.82, *p* <  0.001), hepatocellular carcinoma (Spearman’s correlation, *R* = 0.60, *p* <  0.001), and pancreatic adenocarcinoma (Spearman’s correlation, R = 0.40, *p* <  0.001). Moreover, the co-expression patterns of nuclear FAM83H and nuclear ZNF16 were independent indicators of poor prognosis of gallbladder carcinoma patients. Moreover, the individual and co-expression patterns of nuclear FAM83H and ZNF16 were significantly associated with the prognosis of gallbladder carcinoma patients who received adjuvant chemotherapy. These findings suggest the possibility that the FAM83H-ZNF16 pathway might be involved in the effectiveness of anti-cancer chemotherapy. Therefore, our results suggest that FAM83H and ZNF16 are cooperatively involved in the progression of gallbladder cancers. However, the exact mechanism of the relationship between FAM83H and ZNF16 is unclear. Therefore, further study of the mechanism(s) by which FAM83H/ZNF16 are involved in gallbladder cancer progression is needed.

The significance of the prognostic value of the expressions of FAM83H and ZNF16 in gallbladder carcinoma patients suggests that blocking of FAM83H-ZNF16 pathway might be a potential therapeutic target. The therapeutic potential of blocking of the FAM83H pathway in human cancer is supported by studies in animal models of hepatocellular carcinoma and osteosarcoma [5, 6]. However, a specific therapeutic agent targeting the FAM83H-ZNF16 pathway has not been developed. Therefore, when considering the prognostic impact of FAM83H/ZNF16 expression in gallbladder carcinomas, further study is needed to find specific therapeutics targeting the FAM83H/ZNF16 pathway. However, a close relationship between FAM83H and oncogenic pathways such as, cellular proliferation and EMT of carcinomas, has been reported [5, 6]. Therefore, using established targeted therapeutic molecules which block cellular proliferation and invasiveness might be beneficial in the treatment of gallbladder carcinoma patients with tumors with high expression of FAM83H/ZNF16. Especially, MYC and the Wnt/β-catenin pathway might be a potential therapeutic targets of gallbladder carcinomas with high expression of FAM83H/ZNF16 because MYC is a transcriptional regulator of FAM83H and FAM83H stabilizes β-catenin [5, 6, 28, 29]. In addition, FAM83H is involved in the invasiveness of cancer cells through the EMT pathway in hepatocellular carcinoma and osteosarcoma [5, 6]. Therefore, EMT-associated receptor tyrosine kinase might be a potential therapeutic target of FAM83H-overexpressing cancers [30]. Furthermore, based on previous reports on the role of FAM83H in the proliferation of cancer cells [5, 6], we have searched the GEPIA database for molecules developed as targeted therapeutic agents that are significantly associated with FAM83H/ZNF expression (Accessed May 2, 2020) [14]. In pancreatic adenocarcinoma, there was significant correlation between the expression of FAM83H mRNA and EGFR mRNA (Pearson correlation, *R* = 0.27, *p* <  0.001), and the expression of ZNF16 mRNA and EGFR mRNA (Pearson correlation, *R* = 0.33, *p* <  0.001) [14]. In cholangiocarcinoma, there was a significant correlation between FAM83H mRNA and ERBB2 mRNA (Pearson correlation, *R* = 0.43, *p* = 0.008) [14]. In addition, there was significant correlation between the expression of FAM83H and BRAF (Pearson correlation, *R* = 0.46, *p* = 0.005), and the expression of ZNF16 mRNA and BRAF mRNA (Pearson correlation, *R* = 0.44, *p* = 0.007) [14]. Therefore, when considering the relationship between FAM83H/ZNF16 and tyrosine kinase- and BRAF-pathways, the inhibitors of the tyrosine kinase receptor and BRAF might be useful for the treatment of the poor prognostic subgroup of gallbladder carcinoma with high expression of FAM83H/ZNF16 [31, 32]. Therefore, additional study is needed to evaluate the effectiveness of established anti-cancer agents targeting tyrosine kinase and BRAF in gallbladder carcinomas. Furthermore, our result suggests the possibility that the FAM83H-ZNF16 pathway might be involved in chemoresistance by affecting the survival of gallbladder carcinoma patients who received adjuvant chemotherapy. In a search of the GEPIA database (Accessed May 2, 2020), mRNA expressions of FAM83H/ZNF16 was significantly associated with PARP1 mRNA expression in cholangiocarcinoma (FAM83H versus PARP1; Pearson correlation, *R* = 0.45, *p* = 0.005, ZNF16 versus PARP1; Pearson correlation, *R* = 0.34, *p* = 0.045) [14]. Therefore, therapeutic agents which disrupt resistance to conventional anticancer therapy, such as PARP inhibitors, might be beneficial in the treatment of the poor prognostic subgroup of gallbladder carcinoma with high expression of FAM83H/ZNF16 [33–35]. In osteosarcoma, inhibition of FAM83H inhibited in vivo growth of KHOS/NP osteosarcoma cells, and the PARP inhibitor, olaparib, potentiated the anticancer effect of doxorubicin in KHOS/NP osteosarcoma cells [6, 22]. Therefore, to support this possibility, additional study is needed to explore the role of the FAM83H-ZNF16 pathway in the PARP-related DNA damage repair pathway.

## Conclusions

In conclusion, we present that the expression of FAM83H and ZNF16 are closely associated, and that high expression patterns of these proteins are significantly associated with shorter survival of gallbladder carcinoma patients. Therefore, FAM83H and ZNF16 might be potential therapeutic targets for gallbladder carcinoma patients, and the expression patterns of FAM83H and ZNF16 might be used as novel prognostic indicators for gallbladder carcinoma patients.

## Data Availability

The datasets generated during and/or analyzed during the current study are available from the corresponding author on reasonable request.

## Abbreviations

95% CI: 95% confidence interval
cFAM83H: cytoplasmic expression of FAM83H
cZNF16: cytoplasmic expression of ZNF16
HR: hazard ratio
nFAM83H: nuclear expression of FAM83H
nZNF16: nuclear expression of ZNF16
OS: Overall survival
RFS: Relapse-free survival
